# Cost-effectiveness of empagliflozin for the treatment of heart failure: a systematic review

**DOI:** 10.3389/fphar.2023.1186579

**Published:** 2023-06-30

**Authors:** Jinyu Liu, Dong Liu, Xuepeng Gong, Anhua Wei, Ruxu You

**Affiliations:** ^1^ Department of Pharmacy, Tongji Hospital, Tongji Medical College, Huazhong University of Science and Technology, Wuhan, Hubei, China; ^2^ Department of Pharmacy, Union Hospital, Tongji Medical College, Huazhong University of Science and Technology, Wuhan, Hubei, China

**Keywords:** empagliflozin, heart failure, economic evaluation, cost-effectiveness, systematic review

## Abstract

**Objective:** This study aims to synthesize evidence on the cost-effectiveness of empagliflozin for heart failure (HF).

**Methods:** MEDLINE, Embase, the Cochrane Library, EconLit, CNKI, Wanfang Data and Chongqing VIP were searched to identify original articles on cost-effectiveness of empagliflozin for HF, and literature surveillance ended on 20 November 2022. The reporting quality of the included articles was determined using the Consolidated Health Economic Evaluation Reporting Standards statement.

**Results:** Of 97 articles identified, 11 studies published from 2020 to 2022 met the inclusion criteria, and the overall quality was accepted. The studies were conducted in 8 countries (China, Japan, Korea, Singapore, Thailand, Australia, United States, and United Kingdom). This body of evidence suggested that add-on empagliflozin was cost effective for HF with reduced ejection fraction (HFrEF) patients compared to standard of care alone in all the related studies including China, Japan, Korea, Singapore, Thailand, and Australia. For HF with preserved ejection fraction (HFpEF) patients, add-on empagliflozin was cost effective in China and Australia, but not in United States and Thailand. For HF with diabetes, add-on empagliflozin was cost effective in United Kingdom. Moreover, the incremental cost-effectiveness ratios (ICER) were lower for patients with diabetes than without in subgroup analysis. In the uncertainty analysis of all included studies, the ICERs were most sensitive to the cost of empagliflozin and cardiovascular mortality, followed by the cost of the standard treatment, hazard ratio of HF hospitalization.

**Conclusion:** add-on empagliflozin for HFrEF might be cost-effective or dominant compared with standard of care alone. However, for HFpEF patients, add-on empagliflozin might be cost-effective in China and Australian, but not cost-effective in United States and Thailand.

## Introduction

Heart Failure (HF), a heterogeneous syndrome characterized by significant morbidity and mortality, poor functional capacity and quality of life, and high costs, affects more than 64 million people worldwide (James et al., 2018; Baman and Ahmad, 2020; Savarese et al., 2022). The overall lifetime healthcare costs due to HF per patient was estimated to be USD $126,819 by a systematic review including 16 international studies from 2004 to 2016 (Lesyuk et al., 2018). Due to the raising prevalence of HF, the economic burden of the disease on healthcare expenditures worldwide is even expected to increase. In the US, the total cost for HF was estimated to be USD $30.7 billion in 2012, with projections suggesting a significant increase in costs to USD $69.8 in 2030 (Virani et al., 2021). Therefore, it is imperative to undertake economic evaluation of the therapies for HF to reduce its social and economic burden.

Treatment for HF depends on its cause, symptoms, and ejection fraction, a measure of the heart’s squeezing function. Historically, the standard of care (SoC) for HF is standard heart failure device and drug therapy, which included diuretics, angiotensin-converting enzyme inhibitors, angiotensin II receptor blockers, and β-blockers. Recently, several clinical trials have confirmed that sodium-glucose cotransport 2 (SGLT2) inhibitors could reduced the risk of cardiovascular (CV) death or hospitalization for heart failure (Packer et al., 2020; Packer et al., 2021; Santos-Gallego et al., 2021). Empagliflozin, a SGLT2 inhibitor, is the newest medication approved by the US Food and Drug Administration for HF in 2021 and by the Chinese National Medical Products Administration in 2022. The empagliflozin outcome trial in patients with chronic heart failure (EMPEROR) evaluated that empagliflozin reduced CV mortality or HF hospitalization in patients with HFrEF or HFpEF independently of their glycemic status (Anker et al., 2021; Packer et al., 2021). It is currently the only drug that has been proven to improve the outcome of patients with HFpEF by a large randomized controlled trial. Therefore, empagliflozin is recommended not only for HFrEF, but also for HFpEF by 2022 AHA/ACC/HFSA guideline for the management of HF (Heidenreich et al., 2022).

The clinical effects of empagliflozin for patients with HF are demonstrated. Due to the limitation of healthcare resources, the cost effectiveness of empagliflozin for HF must be considered. Several studies from different countries have evaluated the cost effectiveness of empagliflozin for HF, but there were differences in the study methods and results. Therefore, it is necessary to synthesize these studies so that researchers can quickly obtain more comprehensive economic data. This study is the first systematic review to appraise and synthesize the economic evidence of empagliflozin for HF patients. Our results would provide valuable information to administrators and health workers in making the best decisions.

## Methods

### Literature search

This systematic review was conducted according to the Preferred Reporting Items for Systematic Reviews and Meta-Analyses guidelines (Page et al., 2021). Eligible studies were identified from the following databases: MEDLINE, Embase, the Cochrane Library, and EconLit databases with no language restrictions, and CNKI, Wanfang Data and Chongqing VIP for Chinese-language studies. We restricted the analysis to original articles on cost-effectiveness of empagliflozin for HF, and literature surveillance ended on 20 November 2022. The detailed search strategy was presented in Supplementary Table S1.

### Eligibility criteria

Articles meeting the following criteria were included: 1) target population was patients with HF; 2) empagliflozin intervention was included and comparison was not limited; 3) the original economic evaluation, examined costs with their consequences, and reported incremental cost-effectiveness ratios (ICERs) or incremental cost-utility ratios; 4) complete full-text formats were available. Duplicated literature, reviews, commentaries, conference abstracts, expert opinions, and other secondary research were excluded.

### Study selection

Titles and abstracts were screened against the eligibility criteria and then full-text formats of all potentially relevant publications were obtained and reviewed to decide whether they met the inclusion criteria by two authors. Another discussion could be conducted to resolve discrepancies.

### Reporting quality assessment

The 28-item Consolidated Health Economic Evaluation Reporting Standards (CHEERS) statement (Husereau et al., 2022) was used to appraise the reporting quality of studies. Each item was scored as having met the criteria in full (“1”), not at all (“0”), or not applicable (NA). According to the scores, studies were categorized as good (>75%), moderate (50%–75%), and low (<50%).

### Data extraction and synthesis

We made standardized forms to extract relevant information such as basic information (i.e., authors’ name, target population, intervention and comparison), methods and the main results. A narrative synthesis was used to evaluate the aims, methods, settings, and results of the included studies. If possible, we undertook horizontal comparison of modeling technique, cost perspective, measures of benefit used, ICERs, and results of uncertainty analysis across the studies. For better comparing the results of economic analysis between different currencies, all reported ICERs are converted in US$ for the common price year 2022 using the “CCEMG-EPPI-Centre Cost Converter” Version 1.6 (CCEMG-EPPI-Centre Cost Converter, 2019).

## Result

### Studies identified

**FIGURE 1:**
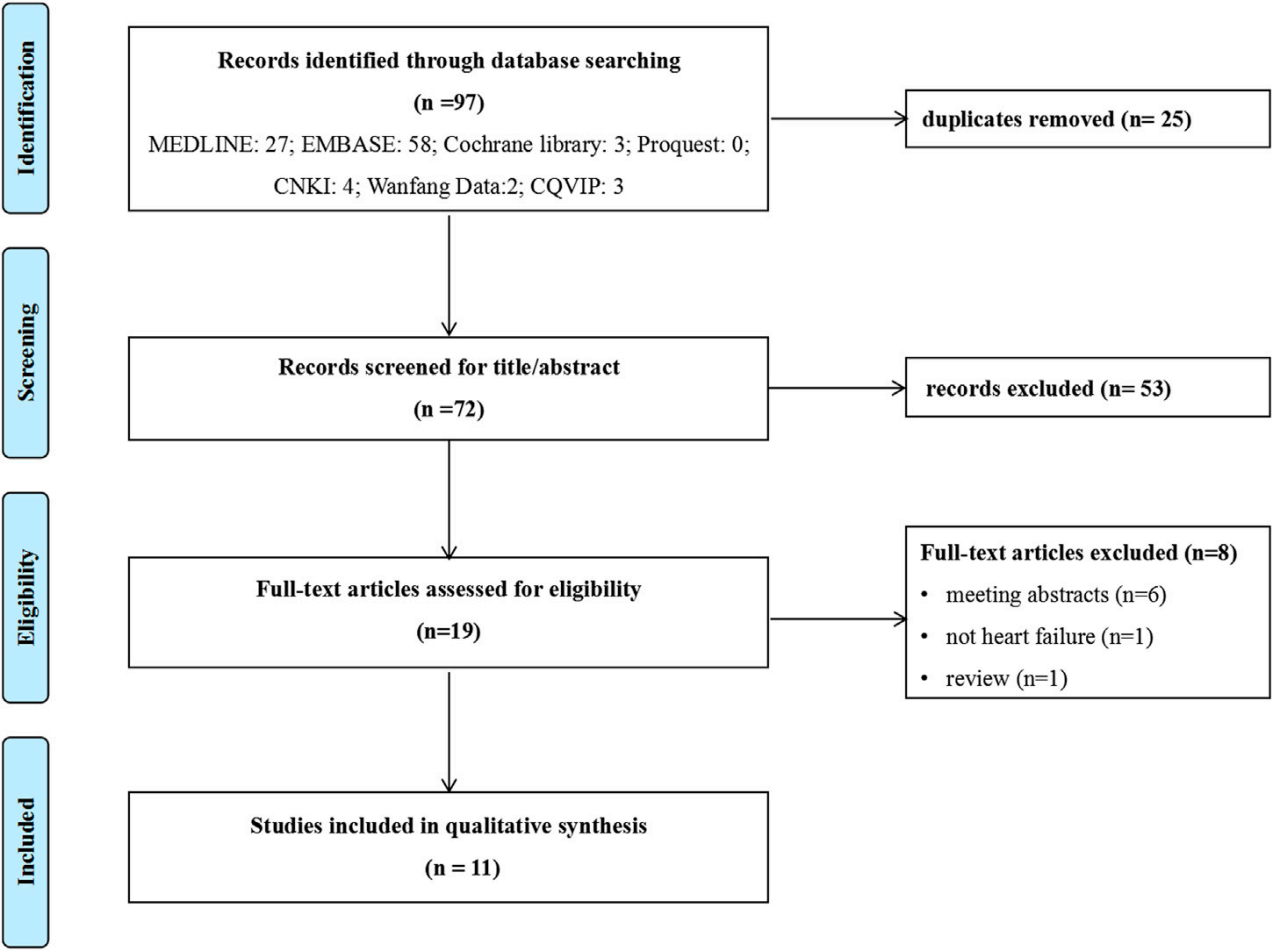
Flowchart of literature search. CNKI: China National Knowledge Infrastructure database; CQVIP: Chongqing VIP database.

Of the 97 potential publications retrieved, 25 were excluded for repetitive publications, and 53 were excluded based on title and abstract. The remaining 19 were retrieved for full-text screening, and 8 were excluded for reasons such as review articles (*n* = 1), not heart failure (*n* = 1), and meeting abstracts (*n* = 6). Finally, 11 publications (Reifsnider et al., 2020; Jiang et al., 2021; Liao et al., 2021; Wan et al., 2022a; Jiang and Xie, 2022; Krittayaphong and Persuwan, 2022; Lin et al., 2022; Lou et al., 2022; Tang and Sang, 2022; Zheng et al., 2022; Zhou et al., 2022) were included in this review, and more details about studies identified were reported in Figure 1.

## Basic characteristics

The general characteristics of the included studies were reported in Table 1. The included studies were conducted in 6 developed countries (United Kingdom, United States, Australia, Korea, Japan, and Singapore) and 2 developing countries (China and Thailand). The Markov model was used in 10 studies, and discrete-event simulation model was used in 1 study. The populations simulated in all the models were based on the basic characteristics of those in the EMPEROR-Preserved study or the EMPEROR-Reduced study. All the included studies compared empagliflozin plus SoC with SoC alone from the healthcare perspective. The time horizons were applied for 10 years or more. Four studies used 1-month Markov cycles, and 6 studies used 3-month Markov cycles.

**TABLE 1:** General characteristics of the included studies.

References	Country	Perspective	Model	Target population	Age	Intervention	Comparison	Cost components	Length of cycle	Time horizon	Discount rate (%)	Health outcomes	Source of effectiveness
Liao et al. (2021)	China (Taiwan), Japan, Korea, Singapore, Thailand, Australia	Healthcare System	Markov	HFrEF	67	EMPA + SoC	SoC	medical costs	1 month	15 years	3	QALY	RCT
Lou et al. (2022)	China	Healthcare system	Markov	HFpEF	72	EMPA + SoC	SoC	direct medical costs	3 months	30 years	5	QALY	RCT
Jiang and Xie (2022)	China	Healthcare system	Markov	HFpEF	66	EMPA + SoC	SoC	the cost of hospitalization for HF, standard therapy, and empagliflflozin	3 months	10 years	5	QALY	RCT
Jiang et al. (2021)	China	Medical and health system	Markov	HFrEF	66	EMPA + SoC	SoC	direct medical costs	3 months	10 years	5	QALY	RCT
Zhou et al. (2022)	Australia	Healthcare system	Markov	HFpEF	72	EMPA + SoC	SoC	direct medical costs	1 month	lifetime	5	QALY	RCT
Lin et al. (2022)	China	Healthcare system	Markov	HFrEF	65	EMPA + SoC	SoC	direct medical costs	3 months	15 years	5	QALY	RCT
Zheng et al. (2022)	United States	Healthcare system	Markov	HFpEF	72	EMPA + SoC	SoC	direct healthcare costs	1 month	lifetime	3	QALY	RCT
Reifsnider et al. (2020)	United Kingdom	Healthcare system	Discrete-event simulation	HF with T2D	-	EMPA + SoC	SoC	direct healthcare costs	-	lifetime	3.5	QALY	RCT
Krittayaphong and Permsuwan (2022)	Thailand	Healthcare system	Markov	HFrEF and HFpEF	60	EMPA + SoC	SoC	direct medical costs	3 months	lifetime	3	QALY	RCT
Tang and Sang (2022)	China	Healthcare system	Markov	HFrEF and HFpEF	65	EMPA + SoC	SoC	direct medical costs	3 months	10 years	5	QALY	RCT
Wan et al. (2022a)	China	Healthcare system	Markov	HFrEF	67	EMPA + SoC	SoC	direct medical costs	1 month	20 years	5	QALY	RCT

*HFrEF,* heart failure with reduced ejection fraction; *HFpEF,* heart failure with preserved ejection fraction; *HF,* heart failure; *T2D,* Type 2 diabetes; *EMPA,* empagliflozin; *SoC,* standard of care; *QALY,* quality-adjusted life-year; *RCT,* randomized controlled trial; *DAPA,* dapagliflozin.

One study was funded by award 1K23HL151672-01 from the National Heart, Lung, and Blood Institute of the National Institutes of Health, one by the Natural Science Foundation of Fujian Province, China and the Health Youth Scientific Research Project of Fujian Province, China, one by Boehringer Ingelheim International GmbH, one by the National Heart Foundation of Australia Fellowship, and one by the National Key Research and Development Program of China. The remaining 6 studies were without funding.

### Reporting quality assessment

All studies have not mentioned three items, which were health economic analysis plan, approach to engagement with patients and others affected by the study, and effect of engagement with patients and others affected by the study respectively. Four studies failed to report characterizing distributional effects (Wan YM. et al., 2022; Jiang and Xie, 2022; Zhou et al., 2022; Reifsnider et al., 2020). The four items mentioned above have been added in the CHEERS statement updated in 2022, therefore the studies did not report well. However, the remaining 24 items were reported sufficiently in all of the included studies, and the included studies were all evaluated as of good quality. More details were summarized in Table 2.

**TABLE 2:** Reporting quality of the economic evaluations (as assessed by the CHEERS statement).

Item no.	Section/item	Liao et al. (2021)	Lou et al. (2022)	Jiang and Xie (2022)	Jiang et al. (2021)	Zhou et al. (2022)	Lin et al. (2022)	Zheng et al. (2022)	Reifsnider et al. (2020)	Krittayaphong and Permsuwan (2022)	Tang and Sang (2022)	Wan et al. (2022a)
1	Title	1	1	1	1	1	1	1	1	1	1	1
2	Abstract	1	1	1	1	1	1	1	1	1	1	1
3	Background and objectives	1	1	1	1	1	1	1	1	1	1	1
4	Health economic analysis plan	0	0	0	0	0	0	0	0	0	0	0
5	Study population	1	1	1	1	1	1	1	1	1	1	1
6	Setting and location	1	1	1	1	1	1	1	1	1	1	1
7	Comparators	1	1	1	1	1	1	1	1	1	1	1
8	Perspective	1	1	1	1	1	1	1	1	1	1	1
9	Time horizon	1	1	1	1	1	1	1	1	1	1	1
10	Discount rate	1	1	1	1	1	1	1	1	1	1	1
11	Selection of outcomes	1	1	1	1	1	1	1	1	1	1	1
12	Measurement of outcomes	1	1	1	1	1	1	1	1	1	1	1
13	Valuation of outcomes	1	1	1	1	1	1	1	1	1	1	1
14	Measurement and valuation of resources and costs	1	1	1	1	1	1	1	1	1	1	1
15	Currency, price date, and conversion	1	1	1	1	1	1	1	1	1	1	1
16	Rationale and description of model	1	1	1	1	1	1	1	1	1	1	1
17	Analytics and assumptions	1	1	1	1	1	1	1	1	1	1	1
18	Characterizing heterogeneity	1	1	1	1	1	1	1	1	1	1	1
19	Characterizing distributional effects	1	1	0	1	0	1	1	0	1	1	0
20	Characterizing uncertainty	1	1	1	1	1	1	1	1	1	1	1
21	Approach to engagement with patients and others affected by the study	0	0	0	0	0	0	0	0	0	0	0
22	Study parameters	1	1	1	1	1	1	1	1	1	1	1
23	Summary of main results	1	1	1	1	1	1	1	1	1	1	1
24	Effect of uncertainty	1	1	1	1	1	1	1	1	1	1	1
25	Effect of engagement with patients and others affected by the study	0	0	0	0	0	0	0	0	0	0	0
26	Study findings, limitations, generalisability, and current knowledge	1	1	1	1	1	1	1	1	1	1	1
27	Source of funding	1	1	1	1	1	1	1	1	1	1	1
28	Conflicts of interest	1	1	1	1	1	1	1	1	1	1	1
Overall quality		Good	Good	Good	Good	Good	Good	Good	Good	Good	Good	Good

Note: “1” meets the quality assessment criteria; “0” does not fully conform to the quality assessment criteria; CHEERS, consolidated health economic evaluation reporting standards.

### Cost-effectiveness analysis

Four studies provided economic evaluation for HFrEF, 4 studies for HFpEF, 2 studies for HFrEF and HFpEF, 1study for HF with type 2 diabetes. The overview of the economic evaluation outcomes are summarized in Table 3.

**TABLE 3:** Overview of economic evaluation outcomes of included studies.

References	Country	Target population	Discount year	Costs (original currency; mean)		QALY		△Cost	△QALY	ICER	ICER (2022 US$ per QALY)	PSA	Uncertainty analysis
				I	C	I	C					WTP (Iterations cost-effectiveness)	
Liao et al. (2021)	China (Taiwan)	HFrEF	2020	**$**79141	**$**71739	9.66	9.30	**$**7402	0.36	**$**20508	$21367.37	**$**25000 (63.4%)	The probability of CV death influenced ICER the most, followed by the probability of non-CV death, monthly costs and utility of stable HF.
												**$**75000 (93.7%)	
	Japan	HFrEF	2020	**$**45210	**$**37664	8.37	8.06	**$**7546	0.31	**$**24046	$25053.62	**$**40137.8 (77.9%)	
												**$**120413.4 (95.6%)	
	Korea	HFrEF	2020	**$**15934	**$**13158	8.37	8.06	**$**2776	0.31	**$**8846	$9216.68	**$**31494.9 (93.6%)	
												**$**94484.7 (96.3%)	
	Singapore	HFrEF	2020	**$**148751	**$**130602	9.02	8.68	**$**18149	0.34	**$**53379	$55615.79	**$**59819.0 (58.1)	
												**$**179457 (94.2%)	
	Thailand	HFrEF	2020	**$**21805	**$**15247	8.11	7.81	**$**6558	0.30	**$**21543	$22445.74	**$**7371.4 (%0)	
												**$**22114.2 (51.9%)	
	Australia	HFrEF	2020	**$**56356	**$**49573	8.63	8.31	**$**6783	0.32	**$**20982	$21861.23	**$**53022.5 (89%)	
												$159067.5 (95.9%)	
Lou et al. (2022)	China	HFpEF	2021	$5423	$4189	4.80	4.68	$1234	0.12	$9881	$10085.41	$37654 (80%)	The cost of EMPA has the largest impact on the ICER.
Jiang and Xie (2022)	China	HFpEF	2021	$5916.50	$4645.23	4.81	4.70	$1271.27	0.11	$11292.06	$11525.67	$12652.5 (52.7%)	The probability of CV death influenced ICER the most, followed by the cost of EMPA, the cost of hospitalization for heart failure, NYHA functional classes, and time horizon
												$37957.5 (67.6%)	
Jiang et al. (2021)	China	HFrEF	2020	$5021.93	$4118.86	3.66	3.53	$903.07	0.13	$6946.69	$7237.78	$11008.07 (55.2%)	The probability of CV death influenced ICER the most, followed by the cost of hospitalization, diabetes status, and time horizon
Zhou et al. (2022)	Australian	HFpEF	2021	A$63218	A$58478	4.97	4.81	A$4740	0.16	A$29202	$20727.20	A$50000 (85%)	The probability of CV death influenced ICER the most, followed by the cost of EMPA and HR of HF hospitalization
Lin et al. (2022)	China	HFrEF	2020	$5220.98	$4873.96	4.86	4.68	$347.02	0.18	$1893.59	$1972.94	$31510.57 (100%)	The major factors affecting the ICER were the cost of EMPA, the cost of the standard treatment, the CV mortality rate in the standard group
Zheng et al. (2022)	United States	HFpEF without CV mortality reduction	2021	$197615	$171357	-	-	$26258	0.06	$437633	$446686.57	$180000 (2.7%)	The results were most sensitive to the monthly cost, quality-of-life benefit, and mortality effect of EMPA.
		HFpEF with CV mortality reduction	2021	$199183	$169438	-	-	$29745	0.17	$174970	$178589.71	$180000 (57.7%)	
Reifsnider et al. (2020)	United Kingdom	HF with T2D	2018	£18,197	£16,829	6.27	5.62	£1368	0.65	£2104	$2124.80	£20,000 (91%)	Variations in the discount rate to costs, the price of EMPA, and the discount rate to QALYs were most influential on the ICER.
Krittayaphong and Permsuwan (2022)	Thai	HFpEF	2021	$929.20	$306.71	4.52	4.47	$622.49	0.05	$12449.8	$12707.36	$4773.27 (11%)	The major factors affecting the ICER were the risk of non-hospitalized CV death, risk of hospitalization in standard treatment, and cost of EMPA.
		HFrEF	2021	$1049.50	$639.68	3.79	3.59	$409.82	0.20	$2049.1	$2091.49	$4773.27 (98%)	The major factors affecting the ICER were the risk of non-hospitalized CV death and cost of EMPA, followed by the risk of non-hospitalized non-CV death
Tang and Sang (2022)	China	HFpEF	2021	$5916.20	$4645.23	4.96	4.85	$1270.97	0.11	$11554.27	$11793.30	$12652.5 (53.1%)	The major factors affecting the ICER were the risk of CV death, followed by the cost of EMPA and the cost of hospitalization for HF.
												$37957.5 (72.2%)	
		HFrEF	2021	$5501.48	$4673.96	4.27	4.12	$827.52	0.15	$5616.80	$5733.00	$12652.5 (59.4%)	
												$37957.5 (72.6%)	
Wan et al. (2022a)	China	HFrEF	2020	¥34,177.91	¥25,864.93	5.74	5.52	¥8 312.98	0.22	¥37,995.94	$11253.80	¥72,447 (58.8%)	The steady-state hospitalization rate of 2 groups was the most important factor affecting the ICER.
												¥217,341 (63.8%)	

*I*, intervention; *C*, comparator; *QALY*, quality-adjusted life year; *ICER*, incremental cost effectiveness ratios; *PSA*, probabilistic sensitivity analyses; *WTP*, willingness-To-pay; *HFrEF*, heart failure with reduced ejection fraction; *CV*, cardiovascular; *HF*, heart failure; *HFpEF*, heart failure with preserved ejection fraction; *EMPA*, empagliflozin; *NYHA*, new york heart association; *HR*, hazard ratio; *T2D*, Type 2 diabetes; *DAPA*, dapagliflozin; *AEs*, Adverse events.

Six studies provided economic evaluation for HFrEF. Four studies conducted in China indicated that adding empagliflozin to SoC was proven to be more cost-effective for HFrEF from a healthcare system perspective. One study conducted in Thailand have the same results as the above studies in China. One study was conducted in China (Taiwan), Australia, Korea, Singapore, Japan, and Thailand. The results showed that adding empagliflozin to SoC for HFrEF was expected to be a cost-effective option, and the probabilities were highest in Korea, lowest in Thailand.

Six studies provided economic evaluation for HFpEF. Three studies were conducted in China, and suggested that the adding empagliflozin to SoC for HFpEF was cost-effective in healthcare systems. One study in Australian suggested that adding empagliflozin is likely to be cost-effective in the healthcare setting. One study in USA suggested that adding empagliflozin provides low economic value compared with SoC for HFpEF. However, the ICER was lower for HFpEF with CV mortality reduction than without. The last study was conducted in Thailand, and suggested that empaglifozin was not a cost-effective add-on treatment for HFpEF. In total, the ICERs were higher for HFpEF than for HFrEF.

### Subgroup analysis

Subgroup analysis was performed according to the different states of diabetes in 3 studies (Jiang et al., 2021; Lin et al., 2022; Zheng et al., 2022), revealing that empagliflozin had similar cost-effectiveness among patients with and without diabetes, and empagliflozin was more cost effective in HErEF patients with diabetes. The details were shown in Table 4. Subgroup analysis was also performed across EF strata and HF-related health status among HErEF patients in 1 study (Zheng et al., 2022), indicating that the ICER was slightly lower for patients with EF less than 50%, and similar for mildly impaired HF and moderately impaired HF.

**TABLE 4:** Subgroup analyses of diabetes status.

References	Country	ICER(US$ per QALY)	
		With diabetes	Without diabetes
Jiang et al. (2021)	China	5016.44	10,844.36
Lin et al. (2022)	China	Dominant	2568.15
Zheng et al. (2022)	United States	Without CV: 419,739	Without CV: 454,942
		With CV: 162,334	With CV: 188,464

ICER, incremental cost effectiveness ratios; QALY, quality-adjusted life year; CV, cardiovascular.

### Uncertainty analysis

One-way sensitivity analysis and probabilistic sensitivity analyses (PSA) were applied in all the included studies. Six studies indicated that the major factor affecting the ICER was the cost of empagliflozin. Three studies (Reifsnider et al., 2020; Lin et al., 2022; Lou et al., 2022) showed when the cost increased to its upper limit, the ICER was still lower than the WTP threshold. One study (Jiang and Xie, 2022) showed when the cost increased to its upper limit, the ICER was higher than one-time GDP but lower than three-time GDP. One study (Zheng et al., 2022) showed that the monthly cost of empagliflozin would need to drop from $326.69 to $153.56 to meet a WTP threshold of $180 000 per quality-adjusted life-year (QALY). One study (Zhou et al., 2022) showed that empagliflozin was no longer cost-effective if its cost exceeded AUD$110 per month.

Six studies displayed the CV mortality to be the most influential parameter. With the decrease of CV mortality in SoC or the increase of CV mortality in adding empagliflozin, the ICERs got higher. One study (Jiang et al., 2021) showed that the CV mortality in adding empagliflozin and SoC had a great impact on the ICER value, which was far more than three-time GDP. Another study (Tang and Sang, 2022) showed the CV mortality in SoC had similar effect. Two studies (Liao et al., 2021; Jiang and Xie, 2022) showed when the CV mortality increased to its upper limit, the ICERs were higher than one-time GDP but lower than three-time GDP. One study (Zhou et al., 2022) showed that adding empagliflozin was no longer cost-effective if the hazard ratio for CV mortality exceeded 0.99. However, there was a study (Krittayaphong and Permsuwan, 2022) showed that the CV mortality did not change the economic outcome.

## Discussion

Eleven economic evaluations of empagliflozin for the treatment of HF from 8 countries were identified in our systematic review, where turns out that add-on empagliflozin is cost effective in most countries, especially for HFrEF patients. The results are similar to the economics of dagliflozin for HF (Krittayaphong and Permsuwan, 2021; Cohen et al., 2023). Unfortunately, there is currently few economic comparison between SGLT2 for the treatment of HF, and it is difficult to determine which SGLT2 is more economical.

In this review, all reported ICERs of different regional backgrounds were adjusted to 2022 USD and the results of PSA were summarized in Table 3 for more convenient comparison. According to the results, the ICERs varied greatly in different studies and different countries. For HFrEF patients, add-on empagliflozin was cost effective in all of the included countries. However, the highest ICER was in Singapore, which was $55615.79 per QALY (Liao et al., 2021), and the lowest ICER was in China, which was $1972.94 per QALY (Lin et al., 2022). It was mainly because of the huge cost difference. For HFpEF patients, with the exception of the USA (Zheng et al., 2022) and Thailand (Liao et al., 2021), it was considered that empagliflozin was cost effective in the remaining countries. It means that the economic results of one country cannot be applied to another, and several economic evaluations have already demonstrated the variability of cost-effectiveness estimates for drugs in different countries (Mac et al., 2019; Li et al., 2021).

It is worth mentioning that the results still varied despite the studies coming from the same country. The ICERs ($22445.74 per QALY vs. $2091.49 per QALY) differ by 10-fold in two studies (Liao et al., 2021; Krittayaphong and Permsuwan, 2022) for HFrEF patients from Thailand. Since Liao et al. did not list specific cost data, there was no way to analyze the reasons for the difference. There were similar situations in Chinese studies. The ICERs of five studies for HFrEF patients varied greatly. One came from Taiwan, China, with the ICER of $21367.37 per QALY (Liao et al., 2021), and the other four were from Chinese mainland with the lowest ICER of $1972.94 per QALY (Lin et al., 2022). The possible reasons for heterogeneity were mainly derived from differences in costs as well as time horizon. Wu et al. (Wu et al., 2022) got similar results in an economic systematic review of dapagliflozin for HF. Therefore, we should consider the heterogeneity in different regions of the same country when evaluating the economics of empagliflozin for HF patients.

Uncertainty analysis showed that the cost of empagliflozin was the major factor affecting the ICERs. With the implementation of centralized procurement of drugs in China, a lower price of empagliflozin is negotiable, hence empagliflozin in treatment for HF patients will be more cost effective from a Chinese healthcare system perspective.

Similar to the previous studies, some critical elements of economic evaluation were also found in our studies (Wan Y. et al., 2022; Liu et al., 2022). Firstly, the election of the target population is crucial, and it could lead to different economic outcomes. For instance, add-on empagliflozin was evaluated to be dominant for HFrEF patients, but not cost-effectiveness for HFpEF patients in the same study (Krittayaphong and Permsuwan, 2022). Furthermore, subgroup analysis performed in 3 studies indicated that empagliflozin was more cost-effectiveness in HErEF patients with diabetes or HF with CV mortality reduction. Hence, the selection of target population is one of the most critical structures of economic evaluation. Secondly, the comparator is a very important element. One study (Jiang et al., 2021) from China showed that add-on empagliflozin was more cost-effectiveness compared with SoC, but led to more costs and less QALY compared with dapagliflozin. We have previously reached the similar results in an economic systematic review of elbasvir/grazoprevir for chronic hepatitis C (Liu et al., 2022). Thirdly, the country or region selected is extremely crucial. The included analyses were mainly for a certain country, so the economic outcomes must be markedly affected by the healthcare system, and economic levels of the country. Therefore, the applicability and extrapolation of research is limited. It will be necessary to improve the methods, such as constructing multi-level models and identifying a series of appropriate covariates to enhance the applicability and extrapolation. Furthermore, There are some other elements that need to be considered (Vandepitte et al., 2021; Yang et al., 2021).

Despite scientific and systematic methods used to minimize deviations, several limitations should be acknowledged. First, The CHEERS statement used in the systematic review is a guideline reporting tool that can help determine whether the study is well reported, but it is not a methodological quality assessment tool. Second, it is extremely difficult to synthesize these studies due to the possible divergences in backgrounds and methodology such as length of cycle, time horizons, target populations, healthcare systems, and cost components, *etc.* For example, although all the studies considered only the direct medical costs, some studies specified the cost details and others did not, which created a limitation to quantitative analysis or horizontal comparison of the studies. Therefore, we summarized the evidence qualitatively and then interpreted the outcomes cautiously. Third, all the included studies have only considered direct medical costs. In fact, HF will cause tremendous social and economic burden if not treated in time. Therefore, it is necessary to carry out further research from the perspective of the whole society. Last but not least, the populations simulated in all the models were based on the EMPEROR study, which means that the reliability of outcomes may be influenced by publication bias.

## Conclusion

In conclusion, this study is the first systematic review on the cost-effectiveness of empagliflozin for HF. Based on the available evidence, add-on empagliflozin for HFrEF might be dominant or cost-effective compared with SoC, and add-on empagliflozin for HFpEF might be cost-effective in China and Australian, but not cost-effective in USA and Thailand. The ICERs were most sensitive to the cost of empagliflozin and CV mortality. In further economic evaluations of empagliflozin for HF patients, the country epidemiological real-world data should be taken into account in model building and sensitivity analysis.

## Data Availability

The original contributions presented in the study are included in the article/Supplementary Material, further inquiries can be directed to the corresponding author.
