# Supplementary material for: Cost-effectiveness of empagliflozin for the treatment of heart failure: a systematic review
Source: Front Pharmacol. 2023 Jun 30;14:1186579. doi: 10.3389/fphar.2023.1186579 (PMC10348886; doi:10.3389/fphar.2023.1186579)
Supplement: Supplementary file 1 [file Table1.DOCX]

**Table S1** Literature search algorithm

**MEDLINE**

| Search strategy | | Item found |
| --- | --- | --- |
| #1 | Cost-benefit analysis[MeSH] OR (cost*[tiab] AND (benefit[tiab] OR effectiveness[tiab] OR utility[tiab]) OR (marginal[tiab] AND analys*[tiab]) OR minimization OR minimisation) OR ((economic*[tiab] OR pharmacoeconomic*[tiab]) AND (analys*[tiab] OR assessment*[tiab] OR evaluat*[tiab] OR implication[tiab] OR health[tiab])) | 966521 |
| #2 | "BI 10773"[tiab] OR BI10773[tiab] OR BI-10773[tiab] OR Jardiance[tiab] OR empagliflozin[tiab] | 2282 |
| #3 | Heart failure [Mesh] OR (((((("Heart failure"[tiab]) OR " Cardiac Failure "[tiab]) OR " Myocardial Failure "[tiab]) OR " Cardiac dysfunction"[tiab] OR " Heart dysfunction "[tiab]) OR " Myocardial dysfunction "[tiab]) | 266871 |
| #4 | #1 AND #2 AND #3 | 27 |

**EMBASE**

| #1 | 'cost benefit analysis'/exp OR ((cost*:ti,ab,kw AND (benefit:ti,ab,kw OR effectiveness:ti,ab,kw OR utility:ti,ab,kw) OR (marginal:ti,ab,kw AND analys*:ti,ab,kw) OR minimization:ti,ab,kw OR minimisation:ti,ab,kw OR economic*:ti,ab,kw OR pharmacoeconomic*:ti,ab,kw) AND (analys*:ti,ab,kw OR assessment*:ti,ab,kw OR evaluat*:ti,ab,kw OR implication:ti,ab,kw OR health:ti,ab,kw)) | 529818 |
| --- | --- | --- |
| #2 | 'heart failure'/exp OR 'heart failure' OR 'heart failure':ti,ab,kw OR 'cardiac failure':ti,ab,kw OR 'myocardial failure':ti,ab,kw OR 'cardiac dysfunction':ti,ab,kw OR 'heart dysfunction':ti,ab,kw OR 'myocardial dysfunction':ti,ab,kw | 709589 |
| #3 | bi10773:ti,ab,kw OR 'bi 10773':ti,ab,kw OR jardiance:ti,ab,kw OR empagliflozin:ti,ab,kw | 3968 |
| #4 | #1 AND #2 AND #3 | 58 |

**Cochrane library**

| #1 | ‘Cost benefit analysis’/exp OR (cost*:ti,ab,kw AND (benefit OR effectiveness OR utility OR minimization OR minimisation):ti,ab,kw OR (marginal AND analys*):ti,ab,kw) OR ((economic* OR pharmacoeconomic*):ti,ab,kw AND (analys* OR assessment* OR evaluat* OR implication OR health):ti,ab,kw)) | 92975 |
| --- | --- | --- |
| #2 | ‘Heart failure’/exp OR (‘Heart failure’ OR ‘Cardiac Failure’ OR ‘Myocardial Failure’ OR ‘Cardiac dysfunction’ OR ‘Heart dysfunction’ OR ‘Myocardial dysfunction’) :ti,ab,kw | 54289 |
| #3 | (‘BI 10773’ OR BI10773 OR BI-10773 OR Jardiance OR empagliflozin):ti,ab,kw | 1527 |
| #4 | #1 AND #2 AND #3 | 3+19（RCT） |

**Proquest**

| #1 | su(Cost benefit analysis) OR ( (TI,AB(cost*) AND TI,AB(benefit OR effectiveness OR utility) OR TI,AB (marginal AND analys*) OR TI,AB (minimization OR minimisation))OR TI,AB(analys* OR assessment*OR evaluat* OR implication OR health) AND TI,AB(economic* OR pharmacoeconomic*)) | 252996 |
| --- | --- | --- |
| #2 | su(Heart failure) OR TI,AB (‘Heart failure’ OR ‘Cardiac Failure’ OR ‘Myocardial Failure’ OR ‘Cardiac dysfunction’ OR ‘Heart dysfunction’ OR ‘Myocardial dysfunction’) | 34898 |
| #3 | TI, AB( ‘BI 10773’ OR BI10773 OR BI-10773 OR Jardiance OR empagliflozin) | 3 |
| #4 | #1 AND #2 AND #3 | 0 |

**Chinese database:**

**China National Knowledge Infrastructure (CNKI)**

| Search strategy | Item found |
| --- | --- |
| (TKA=心衰 OR TKA=心力衰竭) AND (TKA=费用 OR TKA=成本 OR TKA=经济 OR TKA=负担 OR TKA=卫生支出 OR TKA=卫生费用) AND (TKA=恩格列净) | 4 |

**Chinese database: Wanfang Data**

| Search strategy | Item found |
| --- | --- |
| [全部字段=(心衰 OR 心力衰竭) AND 全部字段=(费用OR 成本OR 经济 OR 负担 OR 卫生支出 OR 卫生费用) AND 全部字段=(恩格列净)](http://med.wanfangdata.com.cn/Paper/Search?q=((((%E4%B8%99%E5%9E%8B%E8%82%9D%E7%82%8E) OR %E4%B8%99%E8%82%9D)) AND ((((((%E8%B4%B9%E7%94%A8) OR %E6%88%90%E6%9C%AC) OR %E7%BB%8F%E6%B5%8E) OR %E8%B4%9F%E6%8B%85) OR %E5%8D%AB%E7%94%9F%E6%94%AF%E5%87%BA))) AND (((%E8%89%BE%E5%B0%94%E5%B7%B4%E9%9F%A6) AND %E6%A0%BC%E6%8B%89%E7%91%9E%E9%9F%A6))) | 2 |

**Chinese database: the Chongqing VIP (CQVIP)**

| Search strategy | Item found |
| --- | --- |
| M=(心衰 OR 心力衰竭) AND M=(费用 OR 成本 OR 经济 OR 负担 OR 卫生支出 OR 卫生费用) AND M=(恩格列净) | 3 |
